# A Network Approach to the Association Between Emotional Regulation and ADHD Symptoms in Adults: Pathways between Difficulties in Emotional Regulation and ADHD Dimensions

**DOI:** 10.1007/s11126-026-10270-x

**Published:** 2026-03-23

**Authors:** Rapson Gomez, Taylor Brown, Daniel Zarate, Stephen Houghton, Vasileios Stavropoulos

**Affiliations:** 1 School of Health and Biomedical Science, RMIT University, 264 Plenty Rd, Mill Park, Melbourne, VIC 3082 Australia; 2 School of Psychology, Faculty of Health, Deakin University, Waurn Ponds, Australia; 3 Institute of Health and Wellbeing, Federation University, Ballarat, Australia; 4 Faculty of Education, University of Western Australia, Perth, Australia; 5 Department of Psychology, National and Kapodistrian University of Athens, Athens, Greece

**Keywords:** ADHD, DERS-36, ALS-18, Adults, Difficulties in emotional regulation, Network analysis, Centrality, Edge weights

## Abstract

Investigating the associations between emotional regulation difficulties and ADHD dimensions, this study employs network analysis to examine the interrelations among emotional regulation dimensions (as measured by the Emotional Regulation Strategies-36; DERS-36), affect lability (assessed using the Affect Lability Scale-18; ALS-18) and ADHD dimensions (Inattention, Hyperactivity, and Impulsivity, based on the Current Symptom Scale; CSS). Reflecting on the complexity of ADHD, this research contributes to the emerging discourse on the potential inclusion of emotional regulation difficulties within the ADHD diagnostic criteria, particularly in adult populations. A total of 532 adults [*n* man = 144; women = 388; mean age (*SD*) = 32.98 years (12.97 years)] from the general community were assessed. The network findings showed that the two highest values for strength centrality were DERS-36 strategies and ALS-18 depression. Within the DERS-36, ALS-18, and ADHD dimensions, 33%, 100%, and 11.11% of edges, respectively, were non-zero after regularisation, indicating significant connections, especially with Inattention. For edges involving the ADHD dimensions, only ALS-18 depression and DERS-36 goal showed non-zero unique associations with IA. This study is the first to examine DERS-36 and ALS-18 subdimensions alongside the three ADHD dimensions within a regularised partial correlation network. The results indicate that only a small number of emotion-related dimensions show direct associations with ADHD dimensions, suggesting selective rather than widespread associations among these constructs.

**Supplementary Information:**

The online version contains supplementary material available at 10.1007/s11126-026-10270-x.

## Introduction

According to the fifth edition of the Diagnostic and Statistical Manual of Mental Disorders (DSM-5; American Psychiatric Association [APA], [1], Attention-Deficit/Hyperactivity Disorder (ADHD) is characterized by two core symptom domains: inattention (IA; nine symptoms) and hyperactivity/impulsivity (HY/IM; nine symptoms). Although the DSM-5-TR groups HY and IM as a single diagnostic domain, substantial empirical evidence indicates that, in adults, hyperactivity and impulsivity reflect partially distinct dimensions [2, 3]. Accordingly, the present study examined three symptom dimensions (IA, HY, and IM) to capture these distinctions more precisely.

Some experts have proposed that difficulties in emotion regulation could be another defining feature of ADHD [4–6], especially in adults [7, 8]. Nonetheless, contemporary evidence indicates that difficulties in emotion regulation and affective lability are associated but conceptually distinct from core ADHD symptomatology [5, 9, 10].

Emotion regulation refers to the processes through which individuals monitor, modify, and respond to emotional experiences in ways that facilitate adaptive functioning [11, 12]. Difficulties in emotion regulation, typically operationalised using the DERS-36, reflect disruptions in these processes across domains such as emotional clarity, impulse control, goal-directed behaviour, and access to effective strategies [13]. A related yet conceptually distinct construct is affective lability, assessed by the ALS-18, which captures rapid and pronounced shifts between emotional states rather than difficulties in regulatory processes [14]. Although these constructs can be correlated and often co-occur in ADHD [5, 15, 16], they reflect different aspects of emotional functioning that may show divergent patterns of association with ADHD symptom dimensions.

### Difficulties in Emotional Regulation, Dysfunctional Emotional Regulation, and Affect Lability

Emotion regulation refers to the processes involved in monitoring, modifying, and responding to emotional states in ways that support adaptive functioning [12, 17]. Difficulties in emotion regulation, operationalised using the DERS-36, reflect disruptions in these processes, including limited access to effective regulation strategies, problems with impulse control, and challenges in goal-directed behaviour during emotional arousal [13]. Affective lability, assessed by the ALS-18, represents a related but conceptually distinct construct that refers to rapid and pronounced shifts between emotional states rather than deficits in regulatory processes [14]. Although these constructs may co-occur and both are relevant within ADHD research [5, 15], they capture different aspects of emotional functioning. Accordingly, in this study, the term “difficulties in emotion regulation” refers specifically to the multidimensional regulatory processes assessed by the DERS-36, whereas “affective lability” refers to emotional instability as assessed by the ALS-18.

Although subscales within the ALS-18 use terms such as depression, anxiety, or anger, these labels describe fluctuations between emotional states, not the persistent mood states characteristic of internalising disorders. Internalising symptoms (e.g., sustained sadness, prolonged worry) reflect chronic negative affect, whereas affective lability reflects variability and rapid shifts across affective states. Clarifying this distinction is essential for interpreting ALS-18 dimensions as indicators of emotional instability rather than markers of internalising psychopathology.

### Dysfunctional Emotional Regulation and Affect Lability in ADHD

A qualitative review by Shaw et al., [9]concluded that 34–70% of adults with ADHD also experience difficulties in emotion dysregulation. Existing studies show that both dysfunctional emotion regulation [9, 18, 19] and affect lability [8, 16, 20, 21] are positively and strongly associated with ADHD. Based on the emotion regulation model proposed by Gross [12], the meta-analysis by Graziano and Garcia [15], which involved 77 studies of children and adolescents (*N* = 32,044), concluded that ADHD was associated with the greatest impairment in affect lability (weighted effect size *d* = 0.95), followed closely by dysfunctional emotion regulation (effect size *d* = 0.80), and to a lesser extent empathy/callous–unemotional traits (weighted effect size *d* = 0.68) and emotion recognition/understanding (weighted effect size *d*= 0.64). Similarly, the meta-analysis by Beheshti et al., [5], which included 13 studies of adults (*N* = 2,535), found that affect lability and dysfunctional emotion regulation have strong associations with ADHD (Hedges’ *g* = 1.30 for affect lability and Hedges’ *g* = 1.17 for dysfunctional emotion regulation). Overall, existing data indicate that among the various dimensions of emotional functioning, ADHD is most strongly associated with affect lability and dysfunctional emotion regulation, with the association being slightly stronger for affect lability.

### Limitations in Existing Findings

Overall, there is substantial evidence linking dysfunctional emotion regulation, affect lability, and ADHD; however, existing research remains limited in several important ways.

First, past studies have typically examined ADHD as a unitary construct rather than differentiating its core symptom dimensions (i.e., IA, HY, and IM in adults). As a result, less is known about whether specific emotional processes demonstrate unique associations with distinct ADHD domains.

Second, although dysfunctional emotion regulation and affect lability are multidimensional constructs [13, 14, 22], prior work has often relied on total or composite scores. This approach may obscure whether only particular facets of emotional functioning are uniquely implicated in ADHD. The DERS-36 comprises six subdimensions (nonacceptance, impulse, goals, awareness, strategies, and clarity), whereas the ALS-18 comprises three (anxiety/depression, depression/elation, and normal/anger). Accordingly, associations with ADHD may be selective rather than uniform across subdimensions.

Consistent with this possibility, individuals with ADHD score higher than healthy controls across all ALS-18 dimensions [8, 23, 24]. Correlational findings further show that all six DERS-36 dimensions are positively associated with IA, while all but awareness are associated with HY/IM [25]. When multivariate approaches are applied, however, these patterns become more specific. Using network analysis, Albesisi and Overton, [25]demonstrated that only clarity and goals were uniquely associated with IA, whereas nonacceptance, goals, impulse, and clarity were uniquely associated with HY. Similarly, Tharaud and Nikolas, [26] reported selective, rather than pervasive, links between emotion regulation subdimensions and ADHD symptoms. These findings suggest that broad bivariate associations may overestimate the specificity of emotional–ADHD relationships.

Third, dysfunctional emotion regulation and affective lability are strongly interrelated. High intercorrelations have been documented within the DERS-36 (with the exception of awareness; [13, 27, 28]), within the ALS-18 [14, 29–31], and across the two measures [29, 32]. Consequently, observed associations with ADHD may reflect shared variance among emotional processes rather than unique contributions. Because most studies have not simultaneously modelled these shared associations, the specificity of previous findings remains unclear.

Although affect lability and difficulties with emotion regulation are sometimes viewed as overlapping constructs (Marwaha et al., 2013), neuroimaging evidence indicates they are distinct. Silvers et al., [33] reported that affect lability was positively associated with heightened amygdala activation during emotional reactivity tasks, whereas emotion regulation difficulties were negatively associated with recruitment of the left inferior frontal gyrus during regulatory control tasks. Given that affect lability reflects rapid, intense, and unstable emotional shifts (a bottom-up process), whereas emotion regulation difficulties concern challenges in modulating these emotions and their behavioural consequences (a top-down process), examining both constructs within the same analytic framework provides a more nuanced understanding of their respective contributions to ADHD.

Taken together, these limitations demonstrate that clarifying how emotional functioning relates to ADHD requires a multivariate approach that simultaneously models multiple emotional subdimensions and ADHD symptom dimensions while controlling for shared variance across all variables. Traditional approaches such as partial correlations or multiple regression cannot achieve this: partial correlations isolate pairwise relations but do not adjust for all relevant shared variance, whereas regression models treat variables as predictors or outcomes without modelling interdependencies among them. Network analysis offers an alternative framework that enables the estimation of unique associations among all dimensions within a single interconnected system.

### Network Analysis and Centrality

Network analysis provides a framework for empirically examining patterns of association among psychological variables within a multivariate system [34, 35]. In these models, variables are represented as nodes, and edges represent statistical associations between nodes, with edge weights indicating their strength. In psychological networks, edges are typically estimated using partial correlations, which capture the unique association between two nodes after controlling for all others.

To improve interpretability and reduce spurious associations due to sampling variability, contemporary network models commonly apply regularisation techniques that shrink small edges toward zero (Epskamp & Fried, 36. Regularised partial correlation networks, such as those estimated using the EBICglasso procedure, yield sparser structures that highlight the most robust associations among variables [37]. As a result, the pattern of edges identified in network models often differs from those obtained using zero-order correlations or regression analyses, offering complementary insights into the organisation of psychological constructs [38].

In addition to estimating edges, network analysis provides centrality indices that quantify how strongly each node is connected to others in the network. In cross-sectional Gaussian graphical models, strength centrality (i.e., the sum of absolute non-zero edge weights connected to a node) is the most commonly interpreted metric [39, 40]. Nodes with higher strength centrality are more strongly interconnected within the network and may occupy structurally prominent positions. Although centrality does not imply causality or temporal influence [41, 42], it offers a descriptive indicator of relative connectedness within the system.

Applied to ADHD and emotional functioning, network analysis enables the simultaneous examination of unique associations among ADHD symptom dimensions, difficulties in emotion regulation, and affective lability. This approach allows identification of which emotional subdimensions remain uniquely connected to specific ADHD symptoms once shared variance across all variables is taken into account, thereby addressing key limitations of prior analytic approaches.

### Aims of the Study

The present study aimed to examine the network of associations among difficulties in emotion regulation (DERS-36 subdimensions), affective lability (ALS-18 subdimensions), and ADHD symptom dimensions (IA, HY, and IM) in a community sample of adults. Regularised partial correlation network analysis was used to estimate unique associations among all included dimensions while accounting for shared variance across the system.

Consistent with evidence that separating HY and IM yields clearer associations in adults [2, 3], IA, HY, and IM were modelled as distinct nodes. The resulting network was examined in terms of overall structure, edge weights, strength centrality, and the stability of these estimates.

Based on prior findings, it was expected that subdimensions within the DERS-36 and ALS-18 would show multiple positive associations with one another, and that only a subset of emotional subdimensions would demonstrate unique associations with ADHD symptom dimensions. However, given the limited theoretical basis for predicting specific subdimension-level pathways, no directional or edge-specific hypotheses were specified.

## Method

### Participants

Adults from the general community aged 18 to 65 years (mean age = 32.98, SD = 12.97) were recruited to participate in the study. Supplementary Table S1 provides background information on the initial group of 532 participants. As shown, the sample comprised 144 men (mean age = 34.00, SD = 13.01) and 388 women (mean age = 32.60, SD = 11.95). There was no significant difference between the gender groups in age, t(530) = 1.104, *p* =.270. The majority of participants were either full-time employed or students, had completed higher (university-level) education, and were in some form of relationship (Supplementary Table S1). A small proportion of the sample self-reported a formal diagnosis of ADHD, comprising 7 men and 10 women (total *n* = 17). One participant (female) self-reported a diagnosis of oppositional defiant disorder (ODD).

Virtually all participants (*N* = 525) in the current study were also involved in another study submitted for publication (Gomez & Houghton, submitted). However, that study examined the incremental validity of difficulties in emotion regulation beyond trait impulsivity in predicting ADHD symptoms of inattention and hyperactivity/impulsivity, as well as oppositional defiant disorder symptoms in adults, and did not involve network analysis.

### Measures

All participants completed a demographic questionnaire assessing age, gender, education, employment status, relationship status, and self-reported diagnoses of ADHD and ODD. They also completed the following self-report questionnaires: the Affect Lability Scale-18 (ALS-18; [14]), the Difficulties in Emotion Regulation Scale-36 (DERS-36; [13]), and the Current Symptom Scale (CSS; [43]).

### The Affect Lability Scale-18 (ALS-18 Oliver and Simons, [14])

The ALS-18 is a self-report measure assessing affect lability, defined as rapid and pronounced shifts between emotional states. It was derived from the original 58-item ALS [22]and comprises three subscales: anxiety/depression, depression/elation, and normal/anger. Items are rated on a four-point Likert scale ranging from 0 (“very uncharacteristic of me”) to 3 (“very characteristic of me”), with higher scores indicating greater affective lability. The ALS-18 has demonstrated good construct, convergent, and factorial validity across clinical and community samples, including adults with ADHD and other forms of psychopathology [24, 29, 30]. Prior research has consistently shown that adults with ADHD score higher than healthy controls across all ALS-18 subdimensions [8, 23, 24], supporting its criterion validity in ADHD research. In the present study, internal consistency was high, with Cronbach’s alpha values of 0.91 (anxiety/depression), 0.91 (depression/elation), and 0.88 (normal/anger).

### Difficulties in Emotional Regulation Strategies- 36 (DERS-36 Gratz and Roemer, [13])

The DERS-36 is a self-report measure assessing multiple dimensions of emotion regulation difficulties. The scale consists of 36 items forming six subscales: nonacceptance of emotional responses, difficulties engaging in goal-directed behaviour, impulse control difficulties, lack of emotional awareness, limited access to emotion regulation strategies, and lack of emotional clarity. Items are rated on a five-point Likert scale ranging from 1 (“almost never”) to 5 (“almost always”), with higher scores indicating greater difficulties in emotion regulation. Several items are reverse coded prior to scoring, in accordance with standard scoring procedures. The DERS-36 has demonstrated strong construct validity, factorial validity, and convergent validity across clinical and non-clinical adult samples [13, 27, 28]. Its multidimensional structure has been replicated consistently, and the scale has shown robust associations with a range of psychopathology, including ADHD, supporting its validity for examining emotion regulation processes in adult populations. In the present study, internal consistency for the DERS-36 subscales was acceptable to excellent, with Cronbach’s alpha values of 0.84 (awareness), 0.84 (clarity), 0.87 (impulse), 0.88 (goal-directed behaviour), 0.93 (nonacceptance), and 0.91 (strategies).

### Current Symptom Scale (CSS Barkley and Murphy, [43])

ADHD symptoms were assessed using the Current Symptom Scale (CSS), a self-report measure comprising the 18 DSM-IV/DSM-IV-TR ADHD symptoms, which correspond closely to DSM-5 ADHD criteria. Participants rated the frequency of each symptom over the previous six months on a four-point Likert scale ranging from 0 (“never or rarely”) to 3 (“very often”), with higher scores reflecting greater symptom severity. The CSS has demonstrated good construct validity, criterion validity, and reliability in adult samples and is commonly used in ADHD research [43, 44]. Consistent with prior evidence indicating improved model fit and interpretability when hyperactivity and impulsivity are treated as separable dimensions in adults [2, 45], inattention (IA), hyperactivity (HY), and impulsivity (IM) were modelled as distinct symptom dimensions. In the present study, internal consistency was good for IA (α = 0.89) and acceptable for HY (α = 0.74) and IM (α = 0.73).

### Procedure

Approval for the study was obtained from the Human Research Ethics Committee of XXX University (blinded for review). Following approval, the study was advertised widely on the university’s noticeboards, social media platforms (e.g., Facebook), the Australian Psychological Society’s website, and general community locations such as bus stops. Participants were recruited online over a two-month period using SurveyMonkey. Respondents accessed the survey by clicking a link that directed them to the questionnaires, the order of which was counterbalanced. Completion of the survey was taken as indication of informed consent. Participants recruited via the XXX University psychology participant pool received research participation credit, whereas all other participants received no incentive for taking part.

### Statistical Procedure

A regularised Gaussian Graphical Model (GGM) was estimated using the network module in JASP version 0.14.1.0 (JASP Team, 2018). In this model, nodes represent variables and edges represent partial correlations conditioned on all other nodes. Edges were estimated using the EBICglasso algorithm, which applies graphical LASSO regularisation alongside the Extended Bayesian Information Criterion to obtain a sparse and interpretable network [37]. Although regularisation reduces the estimation of very small or unstable edges, it does not eliminate the possibility that retained edges may still reflect sampling variation; accordingly, edges should be interpreted cautiously, particularly in cross-sectional networks (Epskamp & Fried, [36]. The gamma hyperparameter was set at 0.5, a value shown to balance parsimony and accuracy in psychological networks (Epskamp & Fried, [36]. Twelve nodes were included in the network, resulting in 66 possible edges, and the sample size (*n*= 532) exceeded this number, supporting the stability of the estimation. Node placement in the visualisation was based on the Fruchterman–Reingold force-directed layout, which arranges nodes by simulating attractive forces between connected nodes and repulsive forces between all nodes; thus, nodes connected by stronger edges tend to appear closer together, although their spatial arrangement has no substantive psychological interpretation [46].

Strength centrality was used as the primary centrality index, given it demonstrated stability and interpretability in cross-sectional GGMs [39, 40]. Other centrality indices, such as betweenness and closeness, have shown limited stability and interpretability in psychological networks, and centrality estimates do not imply causal influence; therefore, centrality indices were interpreted descriptively and with caution [36, 41, 42]. Accordingly, betweenness and closeness centrality were not interpreted substantively and are reported for transparency only. Edge-weight magnitude was interpreted using the effect size guidelines proposed by Christensen and Golino [47]. Network stability and accuracy were assessed using non-parametric bootstrapping with 1,000 resamples. Edge-weight precision was evaluated using 95% bootstrap confidence intervals, and centrality stability was examined using case-dropping subset bootstrapping [36]. Full technical details of the analytic procedure are provided in the Supplementary Materials (see Appendix A).

## Results

### Missing Data, Descriptive Statistics, and Correlations

In total, three participants had missing values for items in the ALS-18. For the network analysis, missing data were handled using the pairwise deletion method, meaning that 529 participants were included in the network model. Supplementary Table S2 shows the mean and standard deviation scores for the DERS-36, ALS-18, and CSS dimensions for these 529 participants. As shown, the two dimensions with the highest mean scores were DERS-36 strategies and DERS-36 awareness.

Supplementary Table S3 presents the intercorrelations of all study dimensions. As shown, except for DERS-36 awareness and DERS-36 goal, all other DERS-36 emotion regulation dimensions correlated significantly and positively with one another. DERS-36 awareness and DERS-36 goal did not correlate significantly. Based on Cohen’s [48] guidelines for interpreting effect sizes (small = 0.10, medium = 0.30, large = 0.50), these correlations were all in the medium or large range.

All DERS-36 subdimensions correlated significantly and positively with IA, with small effect sizes. Except for DERS-36 awareness, all DERS-36 subdimensions also correlated significantly and positively with HY, again with small effect sizes. Additionally, with the exception of DERS-36 awareness, DERS-36 clarity, and DERS-36 nonacceptance, all other DERS-36 subdimensions correlated significantly and positively with IM, with small effect sizes. DERS-36 awareness and DERS-36 clarity showed medium-sized correlations with several other DERS-36 dimensions, whereas most remaining associations (except with DERS-36 goal) were in the small range.

### Network Analysis

Given the aims of the study, the results focus on the network graph (data structure), centrality indices, and edge-weight values. Additionally, the results related to establishing the stability and reliability of the centrality and edge-weight findings are also reported. As there were 12 variables (nodes) in the network model, the number of potential edges was 66. However, the application of Markov Random Fields with regularisation reduced the number of estimated edges to 36 (sparsity = 0.45).

### Overview of Key Findings

To aid interpretation, the most important results of the network analyses are summarised here before presenting full technical details. First, emotional-functioning dimensions showed the strongest and most numerous associations within their respective measures, with relatively few meaningful connections across the DERS-36 and ALS-18. Second, among all emotional subdimensions, only two demonstrated unique associations with ADHD symptoms once shared variance was controlled: ALS-18 depression and DERS-36 goal, both of which were linked exclusively to inattention. Third, no DERS-36 or ALS-18 subdimensions showed retained regularised associations with hyperactivity or impulsivity in the multivariate system. These findings indicate that most emotion-related associations reflect internal structure within each measure, while the links between emotional functioning and ADHD are highly selective and specific to inattention.

### Visualization of the Network

**Fig. 1:**
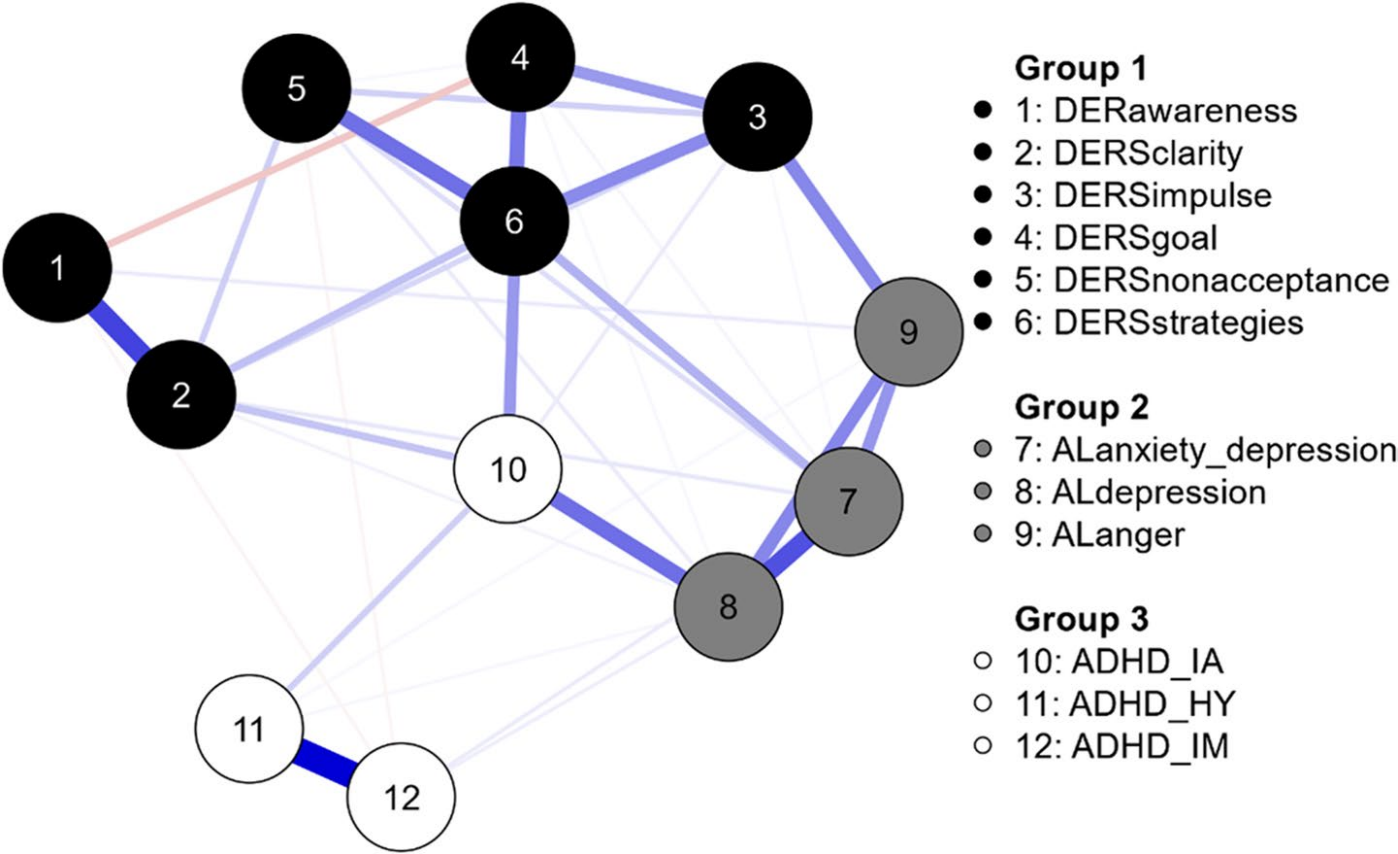
Graphical display of the network. Blue lines represent positive associations, and red lines negative associations. The thickness and brightness of an edge indicate the association strength. The layout is based on the Fruchterman–Reingold algorithm that places the nodes with stronger and/or more connections closer together and the most central nodes into the centre

The relationships among the ADHD nodes in the network are represented visually in Fig. 1. As shown, the DERS-36, ALS-18, and ADHD dimensions are positioned in distinct sections of the network. The ADHD dimension IA appears near the centre, bridging the DERS-36, ALS-18, and ADHD dimensions. The edges in the graph vary in length, intensity, and thickness, indicating variation in the strength of associations between nodes. Apart from the edge between DERS-36 awareness and DERS-36 goal, which was red (negative), all other edges were blue, indicating generally positive associations.

### Centrality of the Nodes in the Network

**Fig. 2:**
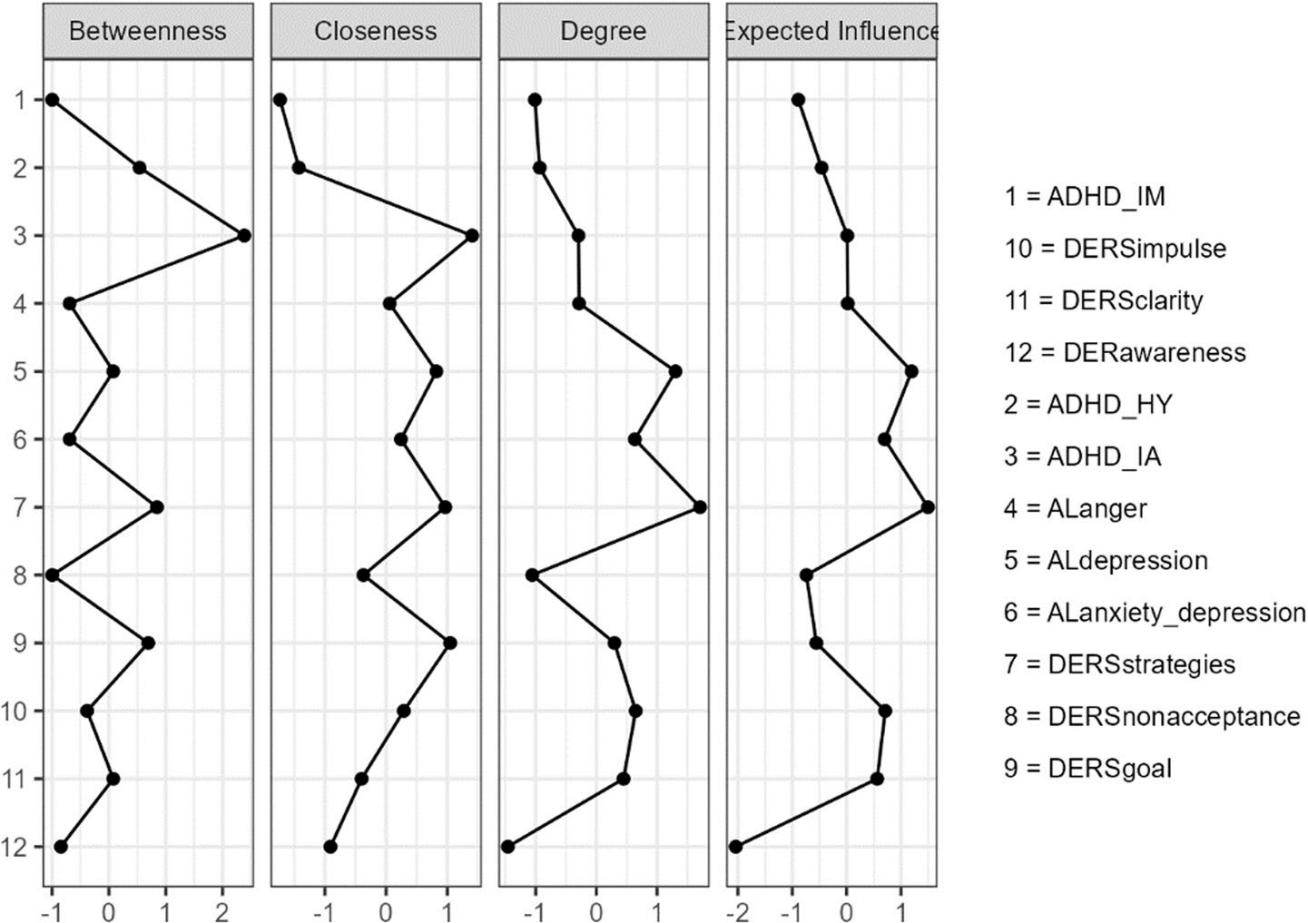
Centrality plots for the nodes in the network. Values shown on the x-axis are standardized z-scores

The centrality of the nodes in the network is shown in Table 1 (reproduced as a figure in Fig. 2). Regarding degree (strength) values, the two nodes with the highest centrality were DERS-36 strategies and ALS-18 depression.

**Table 1:** Centrality indices in the network analysis

Variable	Betweenness	Closeness	Strength	Expected influence
DERS-awareness	-0.85	-0.91	-1.46	-2.04
DERS-clarity	0.08	-0.40	0.45	0.56
DERS-impulse	-0.38	0.29	0.65	0.71
DERS-goal	0.69	1.05	0.30	-0.56
DERS-nonacceptance	-1.00	-0.37	-1.06	-0.74
DERS-strategies	0.85	0.97	1.71	1.50
ALS - anxiety/depression	-0.69	0.24	0.63	0.70
ALS - depression	0.08	0.82	1.30	1.20
ALS - anger	-0.69	0.06	-0.28	0.02
CSS - inattention	2.38	1.41	-0.29	0.01
CSS - hyperactivity	0.54	-1.43	-0.93	-0.46
CSS - impulsivity	-1.00	-1.73	-1.01	-0.89

Higher numbers indicate that the variable is more central to the network. Centrality indices are reported for completeness. Interpretation in the present study focuses on strength centrality, which is considered the most stable and interpretable metric in cross-sectional Gaussian graphical models. Betweenness and closeness centrality have shown limited reliability and validity in psychological networks and should not be interpreted as indicators of causal influence or intervention targets [39, 41, 42]

### Edge Weights for the Difficulties in Emotional Regulation Dimensions in the Network

The weight matrix between nodes is shown in Table 2. When focusing on edges involving the difficulties in emotion regulation dimensions, large positive edge weights were observed between DERS-36 awareness and DERS-36 clarity, and between ALS-18 anxiety/depression and ALS-18 depression. Positive moderate edge weights were present for associations involving DERS-36 impulse with DERS-36 strategies and ALS-18 anger; DERS-36 goal with DERS-36 strategies; DERS-36 goal with DERS-36 nonacceptance; and ALS-18 depression with ALS-18 anger. Positive small edge weights were found for DERS-36 impulse with DERS-36 goal; DERS-36 strategies with ALS-18 anxiety/depression; and ALS-18 anxiety/depression with ALS-18 anger. Across the possible 55 (10 + 9 × 10/2) edges involving the difficulties in emotion regulation dimensions, only 10 (18.18%) could be considered substantively important (i.e., small, moderate, or large in effect size). Of these 10 edges, eight occurred within the same measure (five within the DERS-36 and three within the ALS-18), while only two edges (11.11% of the 18 possible cross-measure edges) connected dimensions across the DERS-36 and ALS-18.

To clarify whether the structure of emotion-related associations in the full network was influenced by the inclusion of ADHD dimensions, and to evaluate the robustness of the emotional-functioning edges, additional post hoc networks were estimated. These supplementary models were designed to determine whether the pattern of connections among DERS-36 and ALS-18 dimensions persisted when examined independently of the broader combined network.

Given these aims, separate post hoc networks were estimated for the DERS-36 dimensions alone, the ALS-18 dimensions alone, and the combined DERS-36 and ALS-18 dimensions. The edge weights for these models are presented in Supplementary Tables S4, S5 and S6. Across all models, the pattern of edge weights closely resembled that observed in the initial full network, indicating that associations among emotional-functioning dimensions were not substantially altered by the inclusion or exclusion of ADHD symptom dimensions.

**Table 2:** Edge Weights in the Network Analysis ADHD Dimensions for IA, HY and IM

Variable	1	2	3	4	5	6	7	8	9	10	11	12
DERS-awareness (1)	0.00	0.43	0.00	− 0.13	0.00	0.00	0.00	0.00	0.04	0.00	0.00	− 0.02
DERS-clarity (2)		0.00	0.09	0.00	0.11	0.14	0.05	0.03	0.00	0.13	0.00	0.00
DERS-impulse (3)			0.00	0.23	0.11	0.26	0.01	0.00	0.27	0.04	0.00	0.00
DERS-goal (4)				0.00	0.02	0.31	0.02	0.02	0.00	0.23	0.00	0.00
DERS-nonacceptance (5)					0.00	0.33	0.07	0.04	0.00	0.00	0.00	− 0.02
DERS-strategies (6)						0.00	0.18	0.00	0.00	0.00	0.00	0.00
ALS-anxiety/depression (7)							0.00	0.40	0.24	0.00	0.00	0.04
ALS-depression (8)								0.00	0.27	0.33	0.02	0.04
ALS-anger (9)									0.00	0.00	0.02	0.00
ADHD-IA (10)										0.00	0.11	0.00
ADHD-HY (11)											0.00	0.58
ADHD-IM (12)												0.00

Medium effect size values are underlined, and large values are bold [based on the Christensen and Golino’s [47], effect size guidelines [(negligible ≤ 0.14, small = ≥ 0.15 to < 0.25, moderate ≥ 0.25 to < 0.35, and large ≥ 0.35)]

### Edge Weights for the Difficulties in Emotional Regulation Dimensions with ADHD Dimensions in the Network

 For the edge weights between the difficulties in emotion regulation and the ADHD dimensions, a moderate non-zero regularised edge weight emerged between ALS-18 depression and IA, and a small non-zero regularised edge weight emerged between DERS-36 goal and IA. Thus, across the ADHD dimensions, only two (out of 27; 11.11%) emotion regulation dimensions showed retained regularised associations—specifically, IA with ALS-18 depression and, to a lesser extent, DERS-36 goal.

 Although our primary focus was on the edge weights linking the DERS-36 and ALS-18 dimensions with the three ADHD dimensions (IA, HY, and IM), a post hoc network analysis was also conducted to examine associations involving a two-factor ADHD structure (IA and HY/IM). The edge-weight estimates for this model are shown in Supplementary Table S7. Consistent with the main model, this post hoc analysis again revealed retained non-zero edges only for IA with ALS-18 depression and DERS-36 goal, with no retained regularised associations between HY/IM and any of the emotion regulation dimensions.

### Stability of the Accuracy of Edge Weights and Centrality Strength Index

As shown in Supplementary Figure S1, for the initial network, when the case-dropping bootstrapping method was applied to evaluate the stability of the centrality indices, the stability coefficient for strength (the centrality index used in this study) remained consistently above 0.5 as the sample was gradually reduced to 25% of the original sample. This result indicates adequate stability for the strength centrality index. As shown in Supplementary Figure S2, when the bootstrap 95% non-parametric confidence interval (CI) method was used to evaluate the stability of the edge weights, the CIs were relatively narrow around the estimated edge-weight values, thereby supporting the stability and precision of the edge-weight estimates.

## Discussion

The present study examined the unique associations among difficulties in emotion regulation, affective lability, and the three ADHD symptom dimensions using a regularised partial correlation network. Overall, the findings revealed a far more selective pattern of relationships than those suggested by traditional bivariate correlations. Although bivariate correlations indicated widespread associations between emotional subdimensions and ADHD symptoms, the network analysis revealed only two unique associations with inattention—ALS-18 depression and, to a lesser extent, DERS-36 goal—and no unique associations with hyperactivity or impulsivity. This pattern suggests that many commonly reported links between emotional dysregulation and ADHD symptoms may reflect shared variance across emotional subdimensions rather than distinct relationships with specific ADHD symptom domains.

 Within the emotional-functioning measures, only a small subset of subdimensions demonstrated unique connections, forming tighter clusters within the DERS-36 and ALS-18 rather than across them. This pattern suggests that difficulties in emotion regulation and affective lability, despite being moderately correlated, operate as partially distinct constructs once their shared variance is modelled. The broader network structure showed that the DERS-36, ALS-18, and ADHD symptom dimensions formed three distinct clusters, with inattention positioned at the interface of emotional and ADHD-related symptom dimensions. Together, these findings refine current understanding of how emotional processes relate to ADHD symptoms in adults, highlighting the specificity of associations and the value of multivariate approaches for disentangling overlapping constructs.

### Theoretical Implications of Our Network Findings

Several theoretical models propose that difficulties in emotion regulation are closely linked to ADHD symptoms, particularly in adults [4–8]. If emotional dysregulation were broadly characteristic of ADHD symptoms, one might expect consistent associations across emotion-related dimensions and all ADHD symptom domains. Instead, the present network identified a more selective pattern, in which only ALS-18 depression and, to a lesser extent, DERS-36 goal, were uniquely associated with inattention, while no emotional dimensions showed unique links with hyperactivity or impulsivity. This pattern is consistent with the possibility that emotional difficulties may relate more strongly to inattentive features than to hyperactive–impulsive symptoms in community adults.

These findings do not permit causal inference given the cross-sectional design and the use of regularised partial correlations. Nevertheless, they are broadly consistent with the view that emotion regulation difficulties are related to, yet conceptually distinct from, core ADHD symptoms [5, 9, 10]. Taken together, the results suggest that particular emotional processes, rather than global emotion dysregulation, may be especially relevant for understanding variability in adult ADHD symptom profiles, although this interpretation remains provisional.

The network’s structure was also consistent with this interpretation. The DERS-36, ALS-18, and ADHD dimensions formed distinct clusters, with inattention situated between the emotional-functioning cluster and the other ADHD symptoms. This configuration aligns with prior work suggesting that emotion regulation difficulties and affective lability are separable constructs that may nonetheless show meaningful associations with ADHD symptoms, particularly inattention [5, 15].

Centrality findings offer additional, though descriptive, theoretical insight. DERS-36 strategies and ALS-18 depression showed the highest strength centrality, indicating that limited access to regulatory strategies and depressive affective lability were the most interconnected emotional dimensions within the network. Although centrality indices cannot be interpreted as indicators of causal influences or clinical priority [41, 42], strength centrality is considered the most stable metric in cross-sectional networks [39, 40]. Accordingly, these findings suggest that these emotional processes may occupy structurally prominent positions within the network, rather than implying direct causal or intervention relevance.

The separation of the DERS-36, ALS-18, and ADHD dimensions into distinct clusters should also be interpreted with caution and does not imply that emotion regulation difficulties are unrelated to ADHD symptoms. Several alternative explanations are plausible, including shared measurement structure, method variance, and the strong internal coherence of questionnaire subscales. In addition, because the present sample did not include individuals with a formal ADHD diagnosis, the observed organisation of clusters may reflect patterns characteristic of community samples rather than clinically diagnosed populations. As such, the clustering pattern is best interpreted as reflecting multivariate associations and measurement properties, rather than as evidence for or against emotional dysregulation as a defining feature of ADHD symptoms.

### Clinical Implications of our Network Findings

The potential clinical relevance of these findings lies in the observed patterning of emotional processes within the broader ADHD–emotion network rather than in any causal interpretation. Although strength centrality reflects the degree to which a dimension is interconnected within the network, it should not be interpreted as indicating causal influence, clinical importance or therapeutic leverage [41, 42]. Accordingly, any clinical implications drawn from the present findings are necessarily preliminary and hypothesis-generating.

Within this network, limited access to emotion-regulation strategies (DERS-36 strategies) and depressive affective lability (ALS-18 depression) were among the most interconnected emotional dimensions. This pattern does not suggest that these processes should be prioritised as treatment targets, but rather that they may tend to co-occur with a range of other emotional difficulties. From a clinical perspective, this may indicate that these dimensions could be relevant to explore when developing an individualised, formulation-oriented understanding of a client’s difficulties.

The selective associations between inattention and only two emotional dimensions (i.e., ALS-18 depression and, more weakly DERS-36 goal) further suggest that emotional difficulties in community adults may be most salient among those with higher inattentive symptom levels. This does not imply that these emotional processes contribute to inattention, nor that treating them would affect ADHD symptoms. Instead, these associations may simply indicate that, during assessment, clinicians could consider screening for co-occurring emotional instability or difficulties in goal-directed behaviour when individuals present with inattentive concerns.

Importantly, these findings do not support the use of emotion regulation or affective lability measures as diagnostic indicators of ADHD symptoms. Rather, they are more consistent with a formulation-oriented approach, in which emotional dimensions are considered alongside attentional symptoms when contextualising an individual’s presentation. Any potential therapeutic relevance of these emotional processes remains speculative and cannot be inferred from the present cross-sectional network. Future research using clinically diagnosed ADHD samples and longitudinal or experimental designs would be required before such findings could inform treatment selection or intervention planning.

### Research Implications of Our Network Findings

The present findings illustrate how network analysis may offer a complementary perspective on the relationships among emotion regulation difficulties, affective lability, and ADHD symptom dimensions. Unlike traditional approaches that quantify shared variance through correlations or regressions, regularised partial correlation networks aim to isolate associations that remain after conditioning on all other variables. This analytic focus may help explain why the current pattern of associations—particularly the selective links between inattention and only two emotional dimensions—differs from patterns typically observed in correlational studies. These differences are likely to reflect methodological contrasts rather than inconsistencies in the underlying constructs, underscoring the importance of using multiple analytical frameworks when investigating complex psychological phenomena.

Given that the present study relied on a single non-clinical sample and a single modelling approach, should be interpreted cautiously and regarded as preliminary. Nevertheless, the results demonstrate the potential utility of network methods for clarifying how emotional processes and ADHD symptoms relate when shared variance among numerous subdimensions is taken into account. Future research integrating network analysis with correlational, regression-based, or structural modelling approaches may help determine which associations consistently emerge across methods and which are more sensitive to analytic choices, measurement structure, or sample characteristics.

The findings also have implications for the measurement and conceptualisation of emotion regulation. Although the DERS-36 and ALS-18 are widely used across diverse psychopathology domains, the present network suggests that their subdimensions cluster separately from ADHD symptoms and from one another, consistent with conceptualisations of emotion regulation and affective lability as related but non-overlapping constructs. However, this interpretation remains tentative and may be influenced by measurement structure. Ongoing efforts to refine models of emotional functioning, such as meta-analytic work identifying higher-order factors including disengagement, aversive cognitive perseveration, and adaptive engagement [49], may be particularly informative when examined alongside network analytic approaches. Such integrative work could help clarify which components of emotional functioning show robust associations with ADHD symptoms across contexts and which reflect broader emotional vulnerability factors.

Ultimately, longitudinal, experimental, and multi-method studies will be required to determine the temporal ordering and potential mechanisms linking emotional processes and ADHD symptoms. The present findings provide a descriptive, hypothesis-generating foundation for this future work, but do not allow conclusions regarding causal pathways, developmental processes, or intervention targets.

## Study Limitations

Several limitations of the current study warrant consideration. First, although network models are often motivated by theoretical frameworks that conceptualise symptoms as mutually interacting systems [34], the present analysis is based on cross-sectional data. Therefore, the estimated edges represent conditional associations only and cannot be interpreted as causal influences or temporal processes. Additionally, although several centrality indices were computed, only strength centrality was interpreted, as other metrics (e.g., betweenness, closeness) have demonstrated limited stability and unclear validity in cross-sectional psychological networks. As such, centrality findings should be viewed as descriptive features of the network structure rather than indicators of causal importance. Second, the sample comprised non-clinical adults recruited from the general community, which limits the generalisability of the findings to clinical populations in which symptom severity, comorbidity patterns, and emotional dysregulation profiles may differ.

Third, the study did not assess psychiatric comorbidities or neurodevelopmental conditions that frequently co-occur with ADHD and are known to influence emotional functioning [50]. The absence of these variables may have introduced unmeasured confounding into the network structure. In addition, the study did not collect information on participants’ history of psychological disorders or prior diagnoses. As a result, it is not possible to determine whether previously identified psychological conditions may have influenced patterns of emotional regulation or ADHD symptoms in the network. This lack of diagnostic history further limits the interpretability and generalisability of the findings, particularly in comparison with clinical samples [51–74].

Fourth, all constructs were measured using self-report instruments, which may differ from clinician-rated or multimethod assessments and could influence the strength or pattern of observed associations. Finally, the results are based on a single dataset and one modelling framework; replication across independent samples and complementary analytic approaches is needed to assess the robustness of the network structure. Despite these limitations, the study provides an initial examination of the joint network of adult ADHD symptoms, difficulties in emotion regulation, and affective lability. As such, it offers preliminary insights that may guide future research into the emotional processes relevant to ADHD symptoms.

## Supplementary Information

Below is the link to the electronic supplementary material.

Supplementary Material 1 (SAV 16.9 KB)

Supplementary file 2 (DOCX 419 KB)

## Data Availability

The data used in the analysis is available with the manuscript.

## Declarations

### Confirmation Statement

The authors confirm that this paper has not been either previously published or submitted simultaneously for publication elsewhere.

## References

[1] American Psychiatric Association. (2013). *Diagnostic and statistical manual of mental disorders* (5th ed.). 10.1176/appi.books.9780890425596

[2] Proctor BE, Prevatt F. Confirming the factor structure of attention-deficit/hyperactivity disorder symptoms in college students using student and parent data. J Learn Disabil. 2009;42(3):250–9. 10.1177/0022219408331043. 19218554 10.1177/0022219408331043

[3] Span SA, Earleywine M, Strybel TZ. Confirming the factor structure of attention deficit hyperactivity disorder symptoms in adult, nonclinical samples. J Psychopathol Behav Assess. 2002;24:129–36. 10.1023/A:1015396926356.

[4] Barkley RA, Fischer M. The unique contribution of emotional impulsiveness to impairment in major life activities in hyperactive children as adults. J Am Acad Child Adolesc Psychiatry. 2010;49(5):503–13. 10.1016/j.jaac.2010.01.019. 20431470 10.1097/00004583-201005000-00011

[5] Beheshti A, Chavanon ML, Christiansen H. Emotion dysregulation in adults with attention deficit hyperactivity disorder: a meta-analysis. BMC Psychiatry. 2020;20:120. 10.1186/s12888-020-2442-7. 32164655 10.1186/s12888-020-2442-7 PMC7069054

[6] Retz W, Stieglitz RD, Corbisiero S, Retz-Junginger P, Rösler M. Emotional dysregulation in adult ADHD: what is the empirical evidence? Expert Rev Neurother. 2012;12(10):1241–51. 10.1586/ern.12.109. 23082740 10.1586/ern.12.109

[7] Hirsch O, Chavanon M, Riechmann E, Christiansen H. Emotional dysregulation is a primary symptom in adult Attention-Deficit/Hyperactivity Disorder (ADHD). J Affect Disord. 2018;232:41–7. 10.1016/j.jad.2018.02.007. 29477097 10.1016/j.jad.2018.02.007

[8] Skirrow C, Asherson P. Emotional lability, comorbidity and impairment in adults with attention-deficit hyperactivity disorder. J Affect Disord. 2013;147(1–3):80–6. 10.1016/j.jad.2012.10.011. 23218897 10.1016/j.jad.2012.10.011

[9] Shaw P, Stringaris A, Nigg J, Leibenluft E. Emotion dysregulation in attention deficit hyperactivity disorder. Am J Psychiatry. 2014;171(3):276–93. 10.1176/appi.ajp.2013.13070966. 24480998 10.1176/appi.ajp.2013.13070966 PMC4282137

[10] Soler-Gutiérrez AM, Pérez-González JC, Mayas J. Evidence of emotion dysregulation as a core symptom of adult ADHD: a systematic review. PLoS One. 2023;18(1):e0280131. 10.1371/journal.pone.0280131. 36608036 10.1371/journal.pone.0280131 PMC9821724

[11] Gross JJ. Emotion regulation: current status and future prospects. Psychol Inq. 2015;26(1):1–26. 10.1080/1047840X.2014.940781.

[12] Gross JJ. The emerging field of emotion regulation: an integrative review. Rev Gen Psychol. 1998;2(3):271–99. 10.1037/1089-2680.2.3.27.

[13] Gratz KL, Roemer L. Multidimensional assessment of emotion regulation and dysregulation: development, factor structure, and initial validation of the difficulties in emotion regulation scale. J Psychopathol Behav Assess. 2004;26:41–54. 10.1023/B:JOBA.0000007455.08539.94.

[14] Oliver MN, Simons JS. The affective lability scales: development of a short-form measure. Pers Indiv Differ. 2004;37(6):1279–88. 10.1016/j.paid.2003.12.013.

[15] Graziano PA, Garcia A. Attention-deficit hyperactivity disorder and children’s emotion dysregulation: a meta-analysis. Clin Psychol Rev. 2016;46:106–23. 10.1016/j.cpr.2016.04.011. 27180913 10.1016/j.cpr.2016.04.011

[16] Merwood A, Chen W, Rijsdijk F, Skirrow C, Larsson H, Thapar A, et al. Genetic associations between the symptoms of attention-deficit/hyperactivity disorder and emotional lability in child and adolescent twins. J Am Acad Child Adolesc Psychiatry. 2014;53(2):209–20. 10.1016/j.jaac.2013.11.006. 24472255 10.1016/j.jaac.2013.11.006

[17] Thompson RA. Emotion regulation: a theme in search of definition. Monogr Soc Res Child Dev. 1994. 10.2307/1166137. 7984164

[18] Corbisiero S, Stieglitz RD, Retz W, Rösler M. Is emotional dysregulation part of the psychopathology of ADHD in adults? ADHD Atten Deficit Hyperact Disord. 2013;5:83–92. 10.1007/s12402-012-0097-z. 10.1007/s12402-012-0097-z 23208078

[19] Fayyad J, Sampson NA, Hwang I, Adamowski T, Aguilar-Gaxiola S, Al-Hamzawi A, et al. The descriptive epidemiology of DSM-IV adult ADHD in the world health organization world mental health surveys. ADHD Atten Deficit Hyperact Disord. 2017;9:47–65. 10.1007/s12402-016-0208-3. 10.1007/s12402-016-0208-3 PMC5325787 27866355

[20] Overgaard KR, Oerbeck B, Aase H, Torgersen S, Reichborn-Kjennerud T, Zeiner P. Emotional lability in preschoolers with symptoms of ADHD. J Atten Disord. 2018;22(8):787–95. 10.1177/1087054715576. 25804545 10.1177/1087054715576342

[21] Sobanski E, Banaschewski T, Asherson P, Buitelaar J, Chen W, Franke B, Faraone SV. Emotional lability in children and adolescents with attention deficit/hyperactivity disorder (ADHD): clinical correlates and familial prevalence. J Child Psychol Psychiatry. 2010;51(8):915–23. 10.1111/j.1469-7610.2010.02217.x. 20132417 10.1111/j.1469-7610.2010.02217.x

[22] Harvey PD, Greenberg BR, Serper MR. The affective lability scales: development, reliability, and validity. J Clin Psychol. 1989;45(5):786–93. 2808736 10.1002/1097-4679(198909)45:5<786::aid-jclp2270450515>3.0.co;2-p

[23] El Archi S, Barrault S, Garcia M, Branger S, Maugé D, Ballon N, et al. Adult ADHD diagnosis, symptoms of impulsivity, and emotional dysregulation in a clinical sample of outpatients consulting for a behavioral addiction. J Atten Disord. 2023;27(7):731–42. 10.1177/10870547231161336. 36945199 10.1177/10870547231161336

[24] Weibel S, Micoulaud-Franchi JA, Brandejsky L, Lopez R, Prada P, Nicastro R, Perroud N. Psychometric properties and factor structure of the short form of the Affective Lability Scale in adult patients with ADHD. J Atten Disord. 2019;23(10):1079–89. 10.1177/1087054717690808. 28152669 10.1177/1087054717690808

[25] Albesisi S, Overton PG. Relationship between ADHD-like traits and emotion dysregulation in the adult general population. Adv Neurodev Disord. 2023. 10.1007/s41252-023-00381-y.

[26] Tharaud JB, Nikolas MA. Emotion regulation as a transdiagnostic link between ADHD and depression symptoms: evidence from a network analysis of youth in the ABCD study. Child Adolesc Psychiatry Ment Health. 2025;19(1):e113. 10.1186/s13034-025-00966-6. 10.1186/s13034-025-00966-6 PMC12538765 41121142

[27] Secinti D, Sen E. Reliability and validity of the brief version of the difficulties in emotion regulation scale in a sample of Turkish adolescents. BMC Psychol. 2023;11(1):165. 10.1186/s40359-023-01199-y. 37208768 10.1186/s40359-023-01199-y PMC10199510

[28] Hallion LS, Steinman SA, Tolin DF, Diefenbach GJ. Psychometric properties of the Difficulties in Emotion Regulation Scale (DERS) and its short forms in adults with emotional disorders. Front Psychol. 2018;9:539. 10.3389/fpsyg.2018.00539. 29725312 10.3389/fpsyg.2018.00539 PMC5917244

[29] Contardi A, Imperatori C, Amati I, Balsamo M, Innamorati M. Assessment of affect lability: psychometric properties of the ALS-18. Front Psychol. 2018;9:427. 10.3389/fpsyg.2018.00427. 29651267 10.3389/fpsyg.2018.00427 PMC5885065

[30] Look AE, Flory JD, Harvey PD, Siever LJ. Psychometric properties of a short form of the Affective Lability Scale (ALS-18). Pers Individ Differ. 2010;49(3):187–91. 10.1016/j.paid.2010.03.030. 10.1016/j.paid.2010.03.030 PMC2893358 20606710

[31] Xu S, Li L, Ju Y. Factor structure and psychometric properties of the affective lability scale-short form in Chinese adolescents. Front Psychiatry. 2022;13:881541. 10.3389/fpsyt.2022.881541. 36465311 10.3389/fpsyt.2022.881541 PMC9713808

[32] Burr EK, Dvorak RD, Stevenson BL, Schaefer LM, Wonderlich SA. Ability to tolerate distress moderates the indirect relationship between emotion regulation difficulties and loss-of-control over-eating via affective lability. Eat Behav. 2021;43:101561. 10.1016/j.eatbeh.2021.101561. 34517279 10.1016/j.eatbeh.2021.101561 PMC8629940

[33] Silvers JA, Hubbard AD, Biggs E, Shu J, Fertuck E, Chaudhury S, et al. Affective lability and difficulties with regulation are differentially associated with amygdala and prefrontal response in women with Borderline Personality Disorder. Psychiatr Res Neuroimaging. 2016;254:74–82. 10.1016/j.pscychresns.2016.06.009. 10.1016/j.pscychresns.2016.06.009 PMC4992645 27379614

[34] Borsboom D, Cramer AO. Network analysis: an integrative approach to the structure of psychopathology. Ann Rev Clin Psychol. 2013;9:91–121. 10.1146/annurev-clinpsy-050212-185608. https://www.annualreviews.org/doi/abs/. 23537483 10.1146/annurev-clinpsy-050212-185608

[35] Boschloo L, van Borkulo CD, Rhemtulla M, Keyes KM, Borsboom D, Schoevers RA. The network structure of symptoms of the diagnostic and statistical manual of mental disorders. PLoS ONE. 2015;10(9):e0137621. 10.1371/journal.pone.0137621. 26368008 10.1371/journal.pone.0137621 PMC4569413

[36] Epskamp S, Borsboom D, Fried EI. Estimating psychological networks and their accuracy: a tutorial paper. Behav Res Methods. 2018;50:195–212. 10.3758/s13428-017-0862-1. 28342071 10.3758/s13428-017-0862-1 PMC5809547

[37] Foygel R, Drton M. (2010). Extended Bayesian information criteria for Gaussian graphical models. *Advances in neural information processing systems*, *23*. https://proceedings.neurips.cc/paper/2010/file/072b030ba126b2f4b2374f342be9ed44-Paper.pdf

[38] Epskamp S, Rhemtulla M, Borsboom D. Generalized network psychometrics: combining network and latent variable models. Psychometrika. 2017;82:904–27. 10.1007/s11336-017-9557-x. 28290111 10.1007/s11336-017-9557-x

[39] McNally RJ. Network analysis of psychopathology: controversies and challenges. Annu Rev Clin Psychol. 2021;17:31–53. 10.1146/annurev-clinpsy-081219-092850. 33228401 10.1146/annurev-clinpsy-081219-092850

[40] Santos HP Jr, Kossakowski JJ, Schwartz TA, Beeber L, Fried EI. Longitudinal network structure of depression symptoms and self-efficacy in low-income mothers. PLoS ONE. 2018;13(1):e0191675. 10.1371/journal.pone.0191675. 29360876 10.1371/journal.pone.0191675 PMC5779701

[41] Bringmann LF, Elmer T, Epskamp S, Krause RW, Schoch D, Wichers M, Snippe E. What do centrality measures measure in psychological networks? J Abnorm Psychol. 2019;128(8):892. 10.1037/abn0000446. 31318245 10.1037/abn0000446

[42] Spiller TR, Levi O, Neria Y, Suarez-Jimenez B, Bar-Haim Y, Lazarov A. On the validity of the centrality hypothesis in cross-sectional between-subject networks of psychopathology. BMC Med. 2020;18:1–14. 10.1186/s12916-020-01740-5. 33040734 10.1186/s12916-020-01740-5 PMC7549218

[43] Barkley RA, Murphy KR. Attention-deficit hyperactivity disorder: A clinical workbook. 2nd ed. Guilford Press; 1998.

[44] Gomez R. ADHD and hyperkinetic disorder symptoms in Australian adults: descriptive scores, incidence rates, factor structure, and gender invariance. J Atten Disord. 2016;20(4):325–34. 10.1177/1087054713485206. 23628968 10.1177/1087054713485206

[45] Gomez R, Stavropoulos V, Watson S, Brown T, Chen W. Inter-relationships between ADHD, ODD and impulsivity dimensions in emerging adults revealed by network analysis: extending the ‘trait impulsivity hypothesis.’ Heliyon. 2022;8(10):e10712. 10.1016/j.heliyon.2022.e10712. 36247147 10.1016/j.heliyon.2022.e10712 PMC9561741

[46] Fruchterman TM, Reingold EM. Graph drawing by force-directed placement. Softw Pract Exp. 1991;21(11):1129–64. 10.1002/spe.4380211102.

[47] Christensen AP, Golino H. Estimating the stability of psychological dimensions via bootstrap exploratory graph analysis: a Monte Carlo simulation and tutorial. Psych. 2021;3(3):479–500. 10.3390/psych3030032.

[48] Cohen J. (1988). *Statistical power analysis for the behavioral sciences* (2nd ed.). Erlbaum.

[49] Naragon-Gainey K, McMahon TP, Chacko TP. The structure of common emotion regulation strategies: a meta-analytic examination. Psychol Bull. 2017;143(4):384. 10.1037/bul0000093. 28301202 10.1037/bul0000093

[50] Jogia J, Sharif AH, Nawaz FA, Khan AR, Alawami RH, Aljanahi MA, et al. Comorbidities associated with attention-deficit/hyperactivity disorder in children and adolescents at a tertiary care setting. Glob Pediatr Health. 2022;9:2333794X221076607. 10.1177/2333794X221076607. 35224143 10.1177/2333794X221076607 PMC8864264

[51] Aas M, Pedersen G, Henry C, Bjella T, Bellivier F, Leboyer M, Kahn JP, Cohen RF, Gard S, Aminoff SR, Lagerberg TV, Andreassen OA, Melle I, Etain B. (2015). Psychometric properties of the Affective Lability Scale (54- and 18-item version) in patients with bipolar disorder, first-degree relatives, and healthy controls. *Journal of Affective Disorders 172*, 375–380.10.1016/j.jad.2014.10.028. 10.1016/j.jad.2014.10.028 25451440

[52] Bardeen JR, Stevens EN. Sex differences in the indirect effects of cognitive processes on anxiety through emotion regulation difficulties. Pers Indiv Differ. 2015;81:180–7. 10.1007/s10862-014-9433-2.

[53] Borsboom D. A network theory of mental disorders. World Psychiatry. 2017;16(1):5–13. 10.1002/wps.20375. 28127906 10.1002/wps.20375 PMC5269502

[54] Bringmann LF, Eronen MI. Don’t blame the model: Reconsidering the network approach to psychopathology. Psychol Rev. 2018;125(4):606. 10.1037/rev0000108. 29952625 10.1037/rev0000108

[55] Brockmeyer T, Skunde M, Wu M, Bresslein E, Rudofsky G, Herzog W, et al. Difficulties in emotion regulation across the spectrum of eating disorders. Compr Psychiatry. 2014;55(3):565–71. 10.1016/j.comppsych.2013.12.001. 24411653 10.1016/j.comppsych.2013.12.001

[56] Castro D, Gysi D, Ferreira F, Ferreira-Santos F, Ferreira TB. Centrality measures in psychological networks: a simulation study on identifying effective treatment targets. PLoS One. 2024;19(2):e0297058. 10.1371/journal.pone.0297058. 38422083 10.1371/journal.pone.0297058 PMC10903921

[57] Charak R, Byllesby BM, Fowler JC, Sharp C, Elhai JD, Frueh BC. Assessment of the revised Difficulties in Emotion Regulation Scales among adolescents and adults with severe mental illness. Psychiatr Res. 2019;279:278–83. 10.1016/j.psychres.2019.04.010. 10.1016/j.psychres.2019.04.010 30975439

[58] Dixon-Gordon KL, Bernecker SL, Christensen K. Recent innovations in the field of interpersonal emotion regulation. Curr Opin Psychol. 2015;3:36–42. 10.1016/j.copsyc.2015.02.001.

[59] Epskamp S, Cramer AO, Waldorp LJ, Schmittmann VD, Borsboom D. Qgraph: network visualizations of relationships in psychometric data. J Stat Softw. 2012;48:1–18. 10.18637/jss.v048.i04.

[60] Gomez R, Gomez RM, Winther J, Vance A. Latent profile analysis of working memory performance in a sample of children with ADHD. J Abnorm Child Psychol. 2014;42:1367–79. 10.1007/s10802-014-9878-5. 24824189 10.1007/s10802-014-9878-5

[61] Kalantzi E, Pehlivanidis A, Korobili K, Mantas V, Papageorgiou C. Psychometric properties of the Greek version of Affective Lability Scale-Short Form (ALS-18) in a sample of adults with neurodevelopmental disorders. Psychiatrike= Psychiatriki. 2022;33(3):200–9. 10.22365/jpsych.2022.063. 35255468 10.22365/jpsych.2022.063

[62] Lilly MM, London MJ, Bridgett DJ. Using SEM to examine emotion regulation and revictimization in predicting PTSD symptoms among childhood abuse survivors. Psychol Trauma Theory Res Pract Policy. 2014;6(6):644. 10.1037/a0036460.

[63] Liu L, Chen W, Vitoratou S, Sun L, Yu X, Hagger-Johnson G, Wang Y. Is emotional lability distinct from angry/irritable mood,negative affect, or other subdimensions of oppositional defiant disorder in children with. ADHD? J Atten disorders. 2019;23(8):859–68. 10.1177/108705471562422. 10.1177/1087054715624228 26842831

[64] Love J, Selker R, Marsman M, Jamil T, Dropmann D, Verhagen J, et al. JASP: graphical statistical software for common statistical designs. J Stat Softw. 2019;88(2):1–17. 10.18637/jss.v088.i02.

[65] McNally RJ, Robinaugh DJ, Wu GW, Wang L, Deserno MK, Borsboom D. Mental disorders as causal systems: a network approach to posttraumatic stress disorder. Clin Psychol Sci. 2015;3(6):836–49. 10.1177/2167702614553230.

[66] Nigg JT, Willcutt EG, Doyle AE, Sonuga-Barke EJ. Causal heterogeneity in attention-deficit/hyperactivity disorder: do we need neuropsychologically impaired subtypes? Biol Psychiatry. 2005;57(11):1224–30. 10.1016/j.biopsych.2004.08.025. 15949992 10.1016/j.biopsych.2004.08.025

[67] Opsahl T, Agneessens F, Skvoretz J. Node centrality in weighted networks: generalizing degree and shortest paths. Soc Networks. 2010;32(3):245–51. 10.1016/j.socnet.2010.03.006.

[68] Robinaugh DJ, Hoekstra RH, Toner ER, Borsboom D. The network approach to psychopathology: a review of the literature 2008–2018 and an agenda for future research. Psychol Med. 2020;50(3):353–66. 10.1017/S0033291719003404. 31875792 10.1017/S0033291719003404 PMC7334828

[69] Rodebaugh TL, Tonge NA, Piccirillo ML, Fried E, Horenstein A, Morrison AS, Heimberg RG. Does centrality in a cross-sectional network suggest intervention targets for social anxiety disorder? J Consult Clin Psychol. 2018;86(10):831. 10.1037/ccp0000336. 30265042 10.1037/ccp0000336 PMC6166439

[70] Ruan QN, Chen CM, Yang JS, Yan WJ, Huang ZX. Network analysis of emotion regulation and reactivity in adolescents: identifying central components and implications for anxiety and depression interventions. Front Psychiatry. 2023;14:1230807. 10.3389/fpsyt.2023.1230807. 37867768 10.3389/fpsyt.2023.1230807 PMC10586221

[71] Ruzzano L, Borsboom D, Geurts HM. Repetitive behaviors in autism and obsessive–compulsive disorder: new perspectives from a network analysis. J Autism Dev Disord. 2015;45:192–202. 10.1007/s10803-014-2204-9. 25149176 10.1007/s10803-014-2204-9

[72] Simons JS, Simons RM, O’Brien C, Stoltenberg SF, Keith JA, Hudson JA. PTSD, alcohol dependence, and conduct problems: distinct pathways via lability and disinhibition. Addict Behav. 2017;64:185–93. 10.1016/j.addbeh.2016.08.044. 27619010 10.1016/j.addbeh.2016.08.044 PMC5143199

[73] Surman CB, Biederman J, Spencer T, Miller CA, Petty CR, Faraone SV. Neuropsychological deficits are not predictive of deficient emotional self-regulation in adults with ADHD. J Atten Disord. 2015;19(12):1046–53. 10.1177/108705471347654. 23503813 10.1177/1087054713476548

[74] von Klipstein L, Borsboom D, Arntz A. The exploratory value of cross-sectional partial correlation networks: predicting relationships between change trajectories in borderline personality disorder. PLoS One. 2021;16(7):e0254496. 10.1371/journal.pone.0254496. 34329316 10.1371/journal.pone.0254496 PMC8323921

