# Supplementary material for: A Network Approach to the Association Between Emotional Regulation and ADHD Symptoms in Adults: Pathways between Difficulties in Emotional Regulation and ADHD Dimensions
Source: Psychiatr Q. 2026 Mar 23;97(2):231–51. doi: 10.1007/s11126-026-10270-x (PMC13328232; doi:10.1007/s11126-026-10270-x)
Supplement: Supplementary file 2 — (DOCX 419 KB) [file 11126_2026_10270_MOESM2_ESM.docx]

**Supplementary Materials**

**Appendix A**

**Statistical Procedure**

The network analysis was conducted using the network module in Jeffreys' Amazing Statistics Program (JASP) version 0.14.1.0 (JASP Team, 2018). The module estimates a Gaussian Graphical Model (GGM), in which nodes represent variables and edges represent partial correlations conditioned on all remaining variables. Regularisation is applied using least absolute shrinkage together with the extended Bayesian Information Criterion (EBIC) model selection to produce regularised partial correlation networks (Foygel & Drton, 2010). With the gamma hyperparameter set at .5, this approach provides accurate network estimation and suppresses small, potentially spurious associations (Epskamp & Fried, 2018).

With 12 nodes included in the analysis, the GGM estimated a total of 91 parameters [(13) + (12 × 13/2)] (Leme et al., 2020). The sample size (*n* = 532) exceeded the number of estimated parameters, indicating adequate power for estimating the network (Epskamp & Fried, 2018).

A network graph is produced in ways to make it easy to interpret. Specifically, more similar nodes are positioned closer to each other, and edge connections are coloured so that positive associations are in blue and negative associations are in red. Additionally, stronger relationships have thicker and more denser lines. In the present study, the Fruchterman and Reingold (1991) procedure for network analysis was applied. This procedure uses an algorithm to position the nodes, so that nodes with stronger correlations are placed near the centre, and nodes with weaker correlations are positioned in the periphery.

The commonly reported indices of centrality are betweenness (i.e., the average distance of a node to all other nodes), closeness (i.e., the inverse sum of all the shortest paths), degree (also called strength, which is the number of non-zero edges a particular node has), and expected influence (the sum of edge weights in weighted networks, accounting for both positive and negative edges, thereby providing an understanding of the cumulative influence a node has on a network; Opsahl et al., 2010; Robinaugh et al., 2016). For all indices, higher values indicated more centrality. Although all four centrality indices are reported in this paper, degree or strength centrality indices will be used here for evaluating the centrality of the nodes as it is known to reflect reasonably precise centrality estimates for psychology networks (McNally, 2021; Santos et al., 2018). Also, the deactivation of nodes based on degree, more than the other indices, has been shown to lead to transforming the network structure (Castro et al., 2024).

Concerning edge weights, Christensen and Golino’s (2021) have provided effect size guidelines for interpreting edges: negligible ≤ .14, small = ≥ .15 to < .25, moderate ≥ .25 to < .35, and large ≥ .35. As we used glasso in our network, the networks produced represent the optimal degree of shrinkage, showing only the most important relations (Borsboom & Cramer, 2013; von Klipstein et al., 2021). Thus, all edge weights displayed in the network, regardless of effect sizes, could be considered worthy of interpretation. However, this would potentially lead to the consideration of a large pool of edge weights, making interpretation difficult. To ease this and facilitate clearer interpretation, the current study considered large, moderate, and small effect sizes as especially important. Negligible effect sizes were not considered important and therefore, were not interpreted.

When a network analysis is conducted, it is expected that the stability and reliability (i.e., the likelihood that the network results will be replicated) of the centrality and edge findings are evaluated and reported. For the current study, the stability of the centrality indices was evaluated using the case-dropping bootstrap (Epskamp & Fried, 2018), which examines if the correlation stability coefficients of the centrality indices remain stable after re-estimating the network with fewer cases. Generally, stability coefficients of .7 or higher are desired, although values of above .5 are considered acceptable (Epskamp et al., 2018). The stability and reliability of the edge weights were evaluated using bootstrap 95% non-parametric confidence intervals (CIs), with narrower CIs suggesting a more precise estimation of the edge (Epskamp et al., 2018). Narrower confidence intervals indicate more stable and precise edge-weight estimates, whereas wider intervals reflect lower stability and greater uncertainty in the estimate. Both the stability coefficients of the centrality indices and edge weights were estimated in the study with 1000 bootstraps.

**Supplementary Table S1.**

*Background Information of Participants*

|  | Frequency | Percentage  (when applicable) |
| --- | --- | --- |
| All | 532 | 100% |
| Gender |  |  |
| Men | 144 | 72.9% |
| Women | 388 | 27.1% |
| Age (Mean; SD) | | |
| All | 32.98 (12.97) |  |
| Men | 34.00 (13.01) | *t* (530) = 1.104, p = .270 |
| Women | 32.60 (11.95) |  |
| Background |  | |
| Relationship | | |
| Single | 155 | 29.1 |
| Married | 192 | 36.1 |
| De-facto | 90 | 16.9 |
| Separate | 10 | 1.9 |
| Divorced | 12 | 2.3 |
| Widowed | 1 | .2 |
| In relation but leaving apart | 72 | 13.5 |
| Education | | |
| Primary | 2 | .4 |
| Secondary/High School | 150 | 28.2 |
| TAFE/Trade Certificate | 91 | 17.1 |
| Undergraduate | 172 | 32.3 |
| Postgraduate | 117 | 22.0 |
| Employment | | |
| Student | 171 | 32.1 |
| Unemployed | 14 | 2.6 |
| Retired | 3.6 | .6 |
| Full Time | 193 | 36.3 |
| Casual / Part Time | 151 | 28.4 |

**Supplementary Table S2.**

*Descriptive for all the Dimensions in the Study*

| Variable | Mean | Standard Deviation |
| --- | --- | --- |
| DERS-awareness | 14.99 | 4.93 |
| DERS-clarity | 10.35 | 3.76 |
| DERS-impulse | 11.07 | 4.68 |
| DERS-goal | 13.68 | 4.95 |
| DERS-nonacceptance | 13.01 | 6.05 |
| DERS-strategies | 16.48 | 7.32 |
| ALS-anxiety/depression | 9.01 | 4.07 |
| ALS-depression | 14.50 | 5.85 |
| ALS-anger | 7.68 | 3.42 |
| CSS-Inattention | 5.90 | 5.21 |
| CSS-Hyperactivity | 4.52 | 3.30 |
| CSS-Impulsivity | 1.60 | 1.78 |

**Supplementary Table S3.**

*Intercorrelations of All the Dimensions in the Study*

| Variable | 1 | 2 | 3 | 4 | 5 | 6 | 7 | 8 | 9 | 10 | 11 | 12 |
| --- | --- | --- | --- | --- | --- | --- | --- | --- | --- | --- | --- | --- |
| DER-awareness (1) | - | .49** | .18** | 0.03 | .17** | .21** | .17** | .15** | .21** | .17** | 0.01 | -0.01 |
| DER-clarity (2) |  | - | .50** | .39** | .48** | .54** | .48** | .46** | .43** | .46** | .12** | 0.08 |
| DER-impulse (3) |  |  | - | .65** | .58** | .72** | .60** | .55** | .64** | .52** | .17** | .13** |
| DER-goal (4) |  |  |  | - | .51** | .69** | .53** | .53** | .46** | .57** | .19** | .11* |
| DER-nonacceptance (5) |  |  |  |  | - | .68** | .53** | .49** | .45** | .39** | .14** | 0.07 |
| DER-strategies (6) |  |  |  |  |  | - | .64** | .57** | .54** | .52** | .17** | .12** |
| AL-anxiety/depression (7) |  |  |  |  |  |  | - | .75** | .68** | .53** | .22** | .22** |
| AL-depression (8) |  |  |  |  |  |  |  | - | .69** | .65** | .26** | .24** |
| AL-anger (9) |  |  |  |  |  |  |  |  | - | .50** | .21** | .18** |
| ADHD-IA (10) |  |  |  |  |  |  |  |  |  | - | .28** | .20** |
| ADHD-HY (11) |  |  |  |  |  |  |  |  |  |  | - | .63** |
| ADHD-IM (12) |  |  |  |  |  |  |  |  |  |  |  | - |

***p* < .01; **p*= < .05

**Supplementary Table S4.**

*Edge Weights in the Network Analysis for the DERS-36 Dimensions*

| Variable | 1 | 2 | 3 | 4 | 5 | 6 |
| --- | --- | --- | --- | --- | --- | --- |
| Awareness (1) | 0.00 | 0.44 | 0.00 | -0.10 | 0.00 | 0.00 |
| Clarity (2) |  | 0.00 | 0.15 | 0.01 | 0.13 | 0.18 |
| Impulse (3) |  |  | 0.00 | 0.30 | 0.14 | 0.32 |
| Goal (4) |  |  |  | 0.00 | 0.04 | 0.36 |
| Nonacceptance (5) |  |  |  |  | 0.00 | 0.36 |
| Strategies (6) |  |  |  |  |  | 0.00 |

*Note*. Medium effect size values are underlined, and large values are bold [based on the Christensen and Golino’s (2021), effect size guidelines [(negligible ≤ .14, small = ≥ .15 to < .25, moderate ≥ .25 to < .35, and large ≥ .35)].

**Supplementary Table S5.**

*Edge Weights in the Network Analysis for the ALS-18 Dimensions*

| Variable | 1 | 2 | 3 |
| --- | --- | --- | --- |
| AL-anxiety/depression (1) | 0.00 | 0.53 | 0.34 |
| AL-depression (2) |  | 0.00 | 0.36 |
| AL-anger (3) |  |  | 0.00 |

*Note*. Medium effect size values are underlined, and large values are bold [based on the Christensen and Golino’s (2021), effect size guidelines [(negligible ≤ .14, small = ≥ .15 to < .25, moderate ≥ .25 to < .35, and large ≥ .35)].

**Supplementary Table S6.**

*Edge Weights in the Network Analysis for the DERS-36 and ALS-18 Dimensions*

| Variable | 1 | 2 | 3 | 4 | 5 | 6 | 7 | 8 | 9 |
| --- | --- | --- | --- | --- | --- | --- | --- | --- | --- |
| Awareness (1) | 0.00 | 0.42 | 0.00 | -0.12 | 0.00 | 0.00 | 0.00 | 0.00 | 0.04 |
| Clarity (2) |  | 0.00 | 0.10 | 0.00 | 0.11 | 0.15 | 0.05 | 0.08 | 0.00 |
| Impulse (3) |  |  | 0.00 | 0.25 | 0.11 | 0.26 | 0.02 | 0.00 | 0.27 |
| Goal (4) |  |  |  | 0.00 | 0.02 | 0.32 | 0.02 | 0.11 | 0.00 |
| Nonacceptance (5) |  |  |  |  | 0.00 | 0.32 | 0.07 | 0.05 | 0.00 |
| Strategies (6) |  |  |  |  |  | 0.00 | 0.17 | 0.00 | 0.00 |
| AL-anxiety/depression (7) |  |  |  |  |  |  | 0.00 | 0.43 | 0.24 |
| AL-depression (8) |  |  |  |  |  |  |  | 0.00 | 0.30 |
| AL-anger (9) |  |  |  |  |  |  |  |  | 0.00 |

*Note*. Medium effect size values are underlined, and large values are bold [based on the Christensen and Golino’s (2021), effect size guidelines [(negligible ≤ .14, small = ≥ .15 to < .25, moderate ≥ .25 to < .35, and large ≥ .35)].

**Supplementary Table S7.**

*Edge Weights in the Network Analysis Involving ADHD Dimensions for IA and HY/IM*

| Variable | 1 | 2 | 3 | 4 | 5 | 6 | 7 | 8 | 9 | 10 | 11 |
| --- | --- | --- | --- | --- | --- | --- | --- | --- | --- | --- | --- |
| DERS-awareness (1) | .00 | .43 | .00 | -.13 | .00 | .00 | .00 | .00 | .05 | .00 | -.02 |
| DERS-clarity (2) |  | .00 | .09 | .00 | .11 | .14 | .05 | .03 | .00 | .11 | .00 |
| DERS-impulse (3) |  |  | .00 | .23 | .11 | .26 | .01 | .00 | .27 | .01 | .03 |
| DERS-goal (4) |  |  |  | .00 | .02 | .31 | .02 | .02 | .00 | .21 | -.05 |
| DERS-nonacceptance (5) |  |  |  |  | .00 | .33 | .07 | .04 | .00 | .00 | .00 |
| DERS-strategies (6) |  |  |  |  |  | .00 | .18 | .00 | .00 | .00 | .00 |
| ALS-anxiety/depression (7) |  |  |  |  |  |  | .00 | .39 | .24 | .00 | .04 |
| ALS-depression (8) |  |  |  |  |  |  |  | .00 | .27 | .21 | .10 |
| ALS-anger (9) |  |  |  |  |  |  |  |  | .00 | .00 | .01 |
| ADHD-IA (10) |  |  |  |  |  |  |  |  |  | .00 | .57 |
| ADHD-HY/IM (11) |  |  |  |  |  |  |  |  |  |  | .00 |

*Note*. Medium effect size values are underlined, and large values are bold [based on the Christensen and Golino’s (2021), effect size guidelines [(negligible ≤ .14, small = ≥ .15 to < .25, moderate ≥ .25 to < .35, and large ≥ .35)].

**Supplementary Figure 1.**

*Stability of Central Indices for the Network*


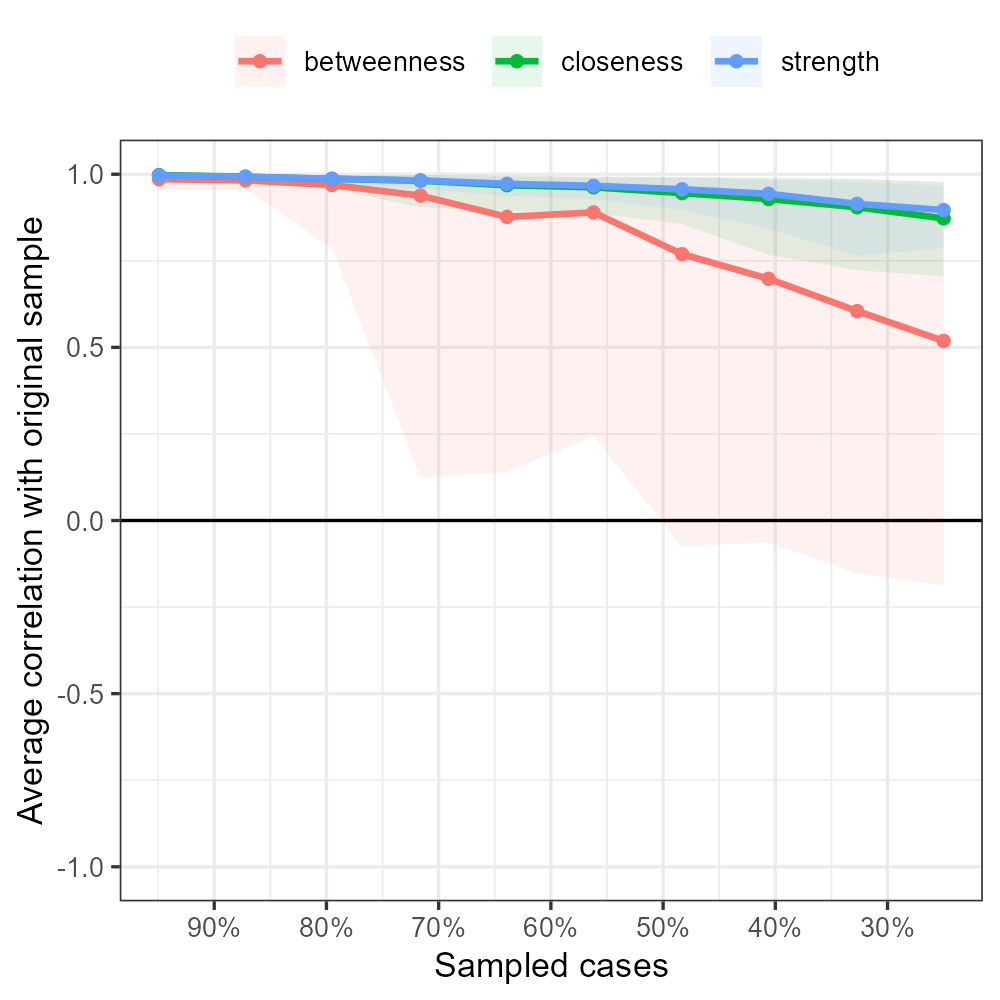


**Supplementary Figure 2.**

*Edge Stability Estimate Using Non-parametric Bootstrapped Estimate for the Network.*


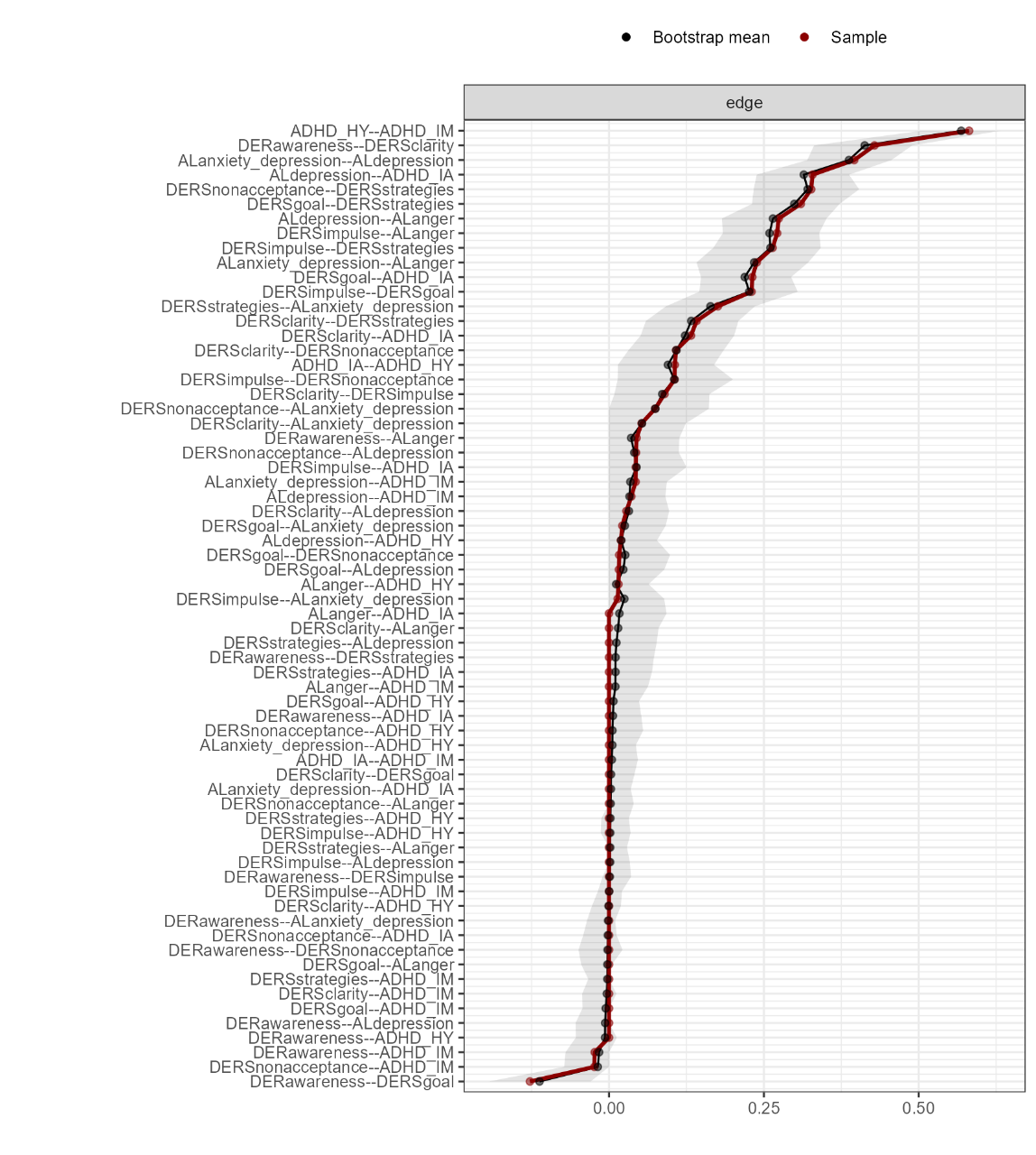


The x-axis represents the edges, while every line on the y-axis represents a specific edge. The red line shows the estimate of the edge stability, and the gray bars the 95% confidence intervals (grey bars) for the estimate.
